# Evaluation of palate area before and after rapid maxillary expansion, using cone-beam computed tomography

**DOI:** 10.1590/2177-6709.24.5.040-045.oar

**Published:** 2019

**Authors:** Carolina Bruder, Cristina Lucia Feijó Ortolani, Tatiana Araújo de Lima, Flavia Artese, Kurt Faltin

**Affiliations:** 1 Universidade Paulista, Programa de Pós-Graduação em Odontologia (São Paulo/SP, Brazil).; 2 Universidade Veiga de Almeida, Curso de Graduação em Odontologia e Fonoaudiologia (Rio de Janeiro/RJ, Brazil).; 3 Universidade do Estado do Rio de Janeiro, Departamento de Odontologia Preventiva e Comunitária (Rio de Janeiro/RJ, Brazil).

**Keywords:** Palatal expansion technique, Cone beam computed tomography, Palate.

## Abstract

**Objective:**

The aim of this study was to estimate the changes in the palate area after rapid maxillary expansion (RME) with the Hyrax expander in growing subjects, using cone beam computed tomography (CBCT).

**Methods:**

Fourteen patients (9 girls and 5 boys; mean age = 11.7 ± 2.4 years) who required RME as part of their orthodontic treatment were included in this study. CBCT records had been taken before RME treatment (T_0_), at the end of active expansion (T_1_) and after a 6-month retention period (T_2_). The CBCT scans were manipulated with Dolphin Imaging^®^ version 11.7 Premium software, in which landmarks were positioned and measured in relation to sagittal, coronal and axial planes, to verify the palate surface area. In addition, linear measurements of the palatal depth and width were assessed. These measurements were compared by using analysis of variance (ANOVA) for repeated measures. A p-value smaller than 0.05 was considered statistically significant.

**Results:**

The palatal surface area and width significantly increased from T_0_ to T_1_, respectively by 9.27% and 9.71%, and both decreased in a non-significant manner from T_1_ to T_2_. The palatal depth had non-significant differences at T_0_, T_1_ and T_2_.

**Conclusions:**

RME promotes a significant gain in the surface area of the palate and an increase in intermolar width. The Hyrax appliance was effective for the treatment of maxillary atresia in growing patients. There was no vertical alteration of the palate. After a 6-month retention period, the maxilla transverse dimension and the surface area of the palate remained stable.

## RESUMO

**Objetivo:**

o objetivo do presente estudo foi estimar a alteração na área do palato após expansão rápida da maxila (ERM) com o expansor Hyrax em indivíduos em crescimento, utilizando tomografia computadorizada de feixe cônico (TCFC).

**Métodos:**

quatorze pacientes (9 meninas e 5 meninos; idade média 11,7± 2,4 anos) que necessitavam de ERM como parte de seu tratamento ortodôntico foram incluídos nesse estudo. Os registros de TCFC foram obtidos antes do tratamento com ERM (T_0_), ao término da expansão ativa (T_1_) e após um período de contenção de 6 meses (T_2_). Os exames de TCFC foram manipulados no *software* Dolphin Imaging^®^ versão 11.7 Premium, no qual os pontos anatômicos foram posicionados e medidos em relação aos planos sagital, coronal e axial, para verificar-se a área de superfície do palato. Além disso, medidas lineares da profundidade e largura do palato foram avaliadas. Essas medidas foram comparadas por análise de variância (ANOVA) para medidas repetidas. Valores de *p*< 0,05 foram considerados estatisticamente significativos.

**Resultados:**

a área de superfície e a largura do palato aumentaram significativamente de T_0_ para T_1_, respectivamente em 9,27% e 9,71%, e ambas diminuíram de forma não significativa de T_1_ para T_2_. A profundidade do palato teve diferenças não significativas em T_0_, T_1_ e T_2_.

**Conclusões:**

a ERM promove ganho significativo na área de superfície do palato e aumento na largura intermolar. O aparelho Hyrax foi efetivo para o tratamento da atrasia maxilar em pacientes em crescimento. Não houve alteração vertical no palato. Após um período de contenção de 6 meses, a dimensão transversal da maxila e a área de superfície do palato permaneceram estáveis.

## INTRODUCTION

Rapid maxillary expansion (RME) is widely used for growing patients in an attempt to correct maxillary constriction.^1^ ^-^ ^3^ This effective procedure increases the width of the maxilla as a result of the separation of the palatine bones through the opening of the midpalatal suture, and buccal inclination of the alveolar bone and molars.^4^ ^,^ ^5^ The activation force is capable of acting not only on the midpalatal suture, but also on the circum-maxillary sutures.^6^ ^,^ ^7^

Traditionally, radiographs are used to identify dentoskeletal changes after RME.^8^ ^-^ ^10^ Through lateral and frontal cephalograms, it is possible to observe that the procedure promotes a downward displacement of the maxilla and an increase in maxillary and nasal widths,^11^ which varies according to the age and severity of the case.^8^

In recent years, three-dimensional images have been used for the same purposes, but with more advantages.^3^ ^,^ ^12^ ^-^ ^16^ Cone-beam computed tomography (CBCT) allows greater resolution, minimal distortion, real size and a low radiation dose, when compared to conventional computed tomography.^17^

Several studies have evaluated the dentoskeletal effects of RME, describing a method to demonstrate the morphological changes of the maxilla that aims to verify, for example, the increase of maxillary width and dental inclinations through CBCT.^1^ ^,^ ^3^ ^,^ ^14^ ^,^ ^17^

Other studies have evaluated changes in the maxilla, such as the morphology and depth of the palate, using dental cast models. Results showed a volumetric and surface increase of the area of the maxilla,which remained stable in the long term; and the palate presented a broader, more harmonic and less deep morphology. In the region of the permanent molars and primary first molars, there was a greater transverse gain.^15^ ^,^ ^16^ ^,^ ^18^

To date, previous studies have evaluated the increase of the maxilla area using dental cast models. The present study aimed to quantify the surface area of the palate of growing patients by means of CBCT using Dolphin Imaging 3D^®^, version 11.7 Premium software (Dolphin Imaging and Management Solutions, Chatsworth, CA, USA). In addition, changes in the depth of the palate and the cross-section of the maxilla were evaluated at the level of the permanent first molar, because of the existence of controversies in the literature.^20^ ^,^ ^22^

## MATERIAL AND METHODS

The records of 14 healthy patients (9 girls and 5 boys) treated with a mean age of 11 years and 7 months ± 2 years and 4 months (minimum age = 8 years and 5 months and maximum age = 14 years and 1 month), met the following requirements: (1) need of RME because of maxillary constriction and posterior bilateral crossbite; (2) Class I molar relationship; (3) lack of adequate space for eruption of permanent canines; (4) cervical vertebrae between CS1 and CS3; and (5) undergone CBCT exams before, immediately after and 6 months after RME. This study was approved by the Ethics and Research Committee of *Universidade Paulista,* under protocol number 1.017.579.

 All patients were treated with an 11-mm Hyrax appliance, with orthodontic bands on the first permanent molars. The appliance was activated with a full turn (0.8 mm) on the day of installation, and parents or guardians were instructed to activate the expander screw with 2/4 turns per day (0.4 mm) for 18 consecutive days until the screw reached an 8-mm opening. After that, the screw was stabilized. The Hyrax appliances were kept passive during a 6-month retention period after expansion.

All the CBCT images from before treatment (T_0_), immediately after expansion (T_1_) and after 6 months of retention with the appliance (T_2_) were obtained and standardized with an i-CAT device (Imaging Sciences International, Hatfield, PA, USA), with the following technical parameters for image acquisition: 120 kV voltage, 8 mA current, 40 seconds exposure, 16 x 8 cm field of view (FOV) and 0.025-mm voxels. 

The data obtained from CBCT were exported in the DICOM (Digital Imaging and Communication in Medicine) format and imported into Dolphin Imaging 3D^®^, version 11.7 Premium software (Dolphin Imaging and Management Solutions, Chatsworth, CA, USA). All the images were manually calibrated by a single operator. 

At the beginning, the volumetric orientation of the maxilla was made with the purpose of standardizing the points to be measured in the different stages (T_0_, T_1_ and T_2_). The tridimensional (3D) images of the maxilla were oriented in the axial, coronal and sagittal planes. In a sagittal view, the ANS (anterior nasal spine) and PNS (posterior nasal spine) were marked by determining the palatal plane oriented parallel to the ground. In this way, the axial plane was created using the palatal plane perpendicular to the midpalatal suture. The coronal plane was determined perpendicular to the axial plane, and the cursor was moved to the distal surface of the second right upper molar, which was chosen as reference. Finally, in order to determine the sagittal plane, the reference was the midpalatal suture and the cursor was moved to this reference perpendicular to the axial plane.

**Figure 1:**
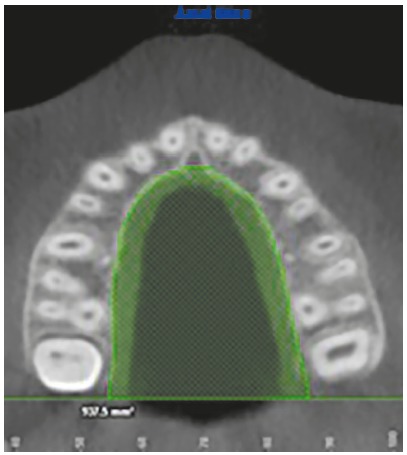
Surface of the palate area (PA).

Using the tool that allows visualization of the three planes (axial, coronal and sagittal), it was lowered along the axial plane until the trifurcation region of the first upper right permanent molar. In order to measure the palatal surface area, the boundaries of the palate were delimited by marking the palatal alveolar ridge from the distal of the first right molar to the distal of the left first molar on the axial plane. This allowed for the measurement of the entire convexity of the palate and then, using the 2D area measurement tool, the area of the palate was determined on the axial plane (Fig 1).

**Figure 2:**
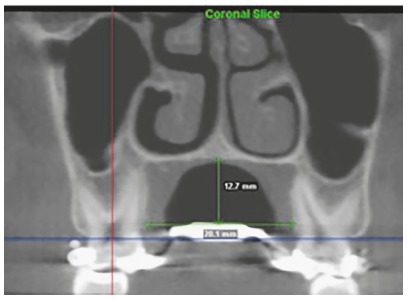
Intermolar width (IMW) and depth of the palate (DP).

In the standard coronal cut, on the first molar region, two linear measurements were selected: intermolar width (IMW) in the region of the lingual bone crest of the first permanent molar from one end to the other (Fig 2), and depth of the palate (DP) measured perpendicular to the IMW at its midpoint to the highest point of the palate (Fig 2).

## STATISTICAL ANALYSIS

For calibration, 10 CBCT images were randomly selected independent of the time interval, and all measurements were performed two times by the same operator within a 2-week interval. All evaluated measurements had an intraclass correlation coefficient (ICC) greater than 93%, indicating the reliability of the measurements.

The Shapiro-Wilk and Bartlett tests showed a normal distribution of the data.

To test the differences of the measurements performed for the 14 patients, at three different moments during observation, a repeated measures analysis of variance, ANOVA, was used with a *post-hoc* test. Significance was set at *p*< 0.05. The descriptive statistics consisted of the means and standard deviations of the surface area of the palate (PA) in mm^2^, intermolar width (IMW) in mm and depth of the palate (DP) in mm.

Based on the sample calculation, this sample with 14 patients, presenting a standard deviation of 3.66, a difference to be detected of 2.7 mm, a significance level of 5% and a two-tailed hypothesis test, has a power of 80%.

## RESULTS

The palatal surface area (PA), intermolar width (IMW) and depth of the palate (DP) from T_0_, T_1_ and T_2_ are summarized in Table 1. The p-values (ANOVA test) for statistically significant differences between the T_0_, T_1_ and T_2_ for all the observed parameters are also listed in Table 1. 

**Table 1:** Comparative values of the measurements of the surface of the palate (PA), Intermolar width (IMW) and depth of the palate (DP) at T_0_, T_1_ and T_2_.

	T_0_	T_1_	T_2_	p-value
	Mean (S.D.)	Mean (S.D.)	Mean (S.D.)	
PA	856.42 (12.40)^A^	935.85 (121.74)^B^	936.21(133.86)^B^	<0.001*
IMW	29.33 (2.72)^A^	32.18 (3.23)^B^	31.64 (3.66)^B^	<0.001*
DP	10.08 (2.73)^A^	10.34 (2.65)^A^	10.27 (2.84)^A^	0.66

Different letters indicate significant differences by repeated measures ANOVA.

* Level of significance p <0.05.

Before treatment (T_0_), the mean palatal surface area (PA) was 856.42 mm^2^, upon removal of the expander (T_1_) it was 935.85 mm^2^, and upon long-term re-examination (T_2_) it was 936.21 mm^2^ (Table 1). Between T_0_ and T_1_, a statistically significant increase in volume (*p*< 0.001) of almost 10% was seen, and a minimum increase in area between T_1_ and T_2_ of 0.1% was not found to be significant.

The intermolar width (IMW) values significantly increased from T_0_ to T_1_, because of the expansion caused by RME: at baseline (T_0_) it was 29.33 mm, and after RME (T_1_) it was 32.18 mm. An increase of almost 10% (*p*< 0.001) was observed from T_0_ to T_1_, whereas the reduction in mm between T_1_ and T_2_ of 1.5% was not found to be significant (Table 1).

However, no statistically significant difference (*p*= 0.66) for depth of the palate (DP) was observed between all the evaluated periods of time. The differences between T_0_ (10.08 mm), T_1_ (10.34 mm) and T_2_(10.27 mm) were minimum, remaining relatively stable during all evaluation periods (Table 1).

The lack of statistically significant differences from T_1_ to T_2_ for all evaluated parameters - palatal surface area (PA), intermolar width (IMW) and depth of the palate (DP) - demonstrate the stability of all measures evaluated during the retention period (Table 1).

## DISCUSSION

The aim of the present study was to evaluate the surface area of the palate before (T_0_), immediately after (T_1_) and six months after the RME (T_2_). A significant increase of 79.43 mm^2^ (9,27%) between T_0_ and T_1_ was observed (*p*< 0.001), remaining stable at T_2_ (passive retention period with the appliance in position). These results, by means of CBCT, corroborate the results of Primozic et al,^18^ who calculated the surface area of children’s palates in the deciduous dentition with an unilateral crossbite (experimental group) and without an unilateral crossbite (control group) using 3D dental models. After treatment, it was concluded that the area of the palate in the crossbite group increased significantly and did not present significant differences when compared to the control group (*p*> 0.05), suggesting that this increase corresponds to the opening of the suture, since the final result of the palate area resembles that of the control group, in which tooth inclination does not occur.

Many authors have already demonstrated the efficacy of RME in the transversal maxillary gain.^4,5,9,18,19,21-24^ However, there are controversies about the amount of this expansion and the structures that provide it. Garrett et al^3^ evaluated 30 patients with a mean age of 13.8 years and observed that after RME, 49% of the expansion was a result of anchoring molar inclination, 13% resulted from alveolar inclination, and 38% resulted from maxillary suture opening. In the present study, the maxillary width in the alveolar bone crest region of the first molar was used as the reference, and the results showed that there was a significant increase, 9.71%, from T_0_ (29.33 mm) to T_1_ (32.18 mm). From T_1_ to T_2_ (31.64 mm), there was a small recurrence (0.54 mm) that may be justified because of the small percentage of alveolar inclination in relation to the maxillary suture opening, as suggested by Garrett et al.^3^

According to some authors,^14^ ^,^ ^17^ the force of the device tips the anchored teeth and their alveolar bone in the same magnitude and direction, but in the long-term, for growing patients, they upright without periodontal damage. However, if the retention time is less than six months, there may be a recurrence, causing a decrease in the transverse width of the maxilla.^20^ In this study, the appliances were kept passive during a 6-month retention period after expansion, as recommended in the literature;^20^ however, a small relapse was observed (0.54 mm).

Lione, Franchi and Cozza^17^ observed an increase of 2.46 mm (on average) in the transverse maxillary dimension in growing patients. Cross and McDonald^9^ also showed a slight increase in the maxillary width, but they suggest that this result was due to the severity of the case and the excessive inclination of the molars. In the present study, there was a small decrease in the intermolar width from T_1_ to T_2_. If the inclination of the alveolar bone that normally occurs because of the increase of the intermolar width contributes to the increase of the area, it should be expected a decrease of the area at T_2_; however, there was a slight increase of the area (0.36 mm^2^) from T_1_ to T_2_. Therefore, this result suggests that the area gain may be directly related to the opening of the midpalatal suture. Thus, it can be affirmed that RME can be indicated for the correction of palatal constriction.

 According to Haas,^1^ ^,^ ^2^ a reduction of the palatal plane is expected after RME; in this way, a decrease in the depth of the palate would also be expected. Ramoglu and Savi,^24^ as well as Woller et al,^8^ observed a slight downward inclination of the palatal plane, but without clinical and statistical significance. The latter concluded that after RME, the opening of the frontonasal, intermaxillary, medial palatine, and zygomaticomaxillary sutures, whose average opening is 1.54 mm, causes the border of this suture to move slightly downward by 0.1 mm and forward by 0.88 mm.

Phatouros and Goonerwardene^21^ showed a slight increase of 0.09 mm in the depth of the palate at the level of the permanent first molar. This increase was statistically insignificant when compared to the control group. In addition, Ladner and Muhl^10^ also observed an increased palate depth attributed to alveolar growth. These results corroborate with the present study. Although the measurements of the depth of the palate (DP) did not show significant differences, from T_0_ to T_1_, an increase of 0.26 mm was observed, what can be attributed to the inclination of the bony crest that is generated by dental inclination.

From T_1_ to T_2_, a decrease of 0.07 mm in DP was observed due to the small recurrence favoring the uprighting of the molars and thus of the bone crest. From T_0_ to T_2_, the increase was 0.19 mm, demonstrating that the palate does not undergo reduction. According to Enlow,^7^ the process of lowering the palate is 50% because of the displacement of the maxilla (associated with the growth of the facial sutures) and 50% because of the new bone formation on the surface of the hard palate and resorption of the nasal cavity floor. This process occurs throughout the entire growth phase; thus, it cannot be said that RME alone promotes palatal retraction, as some studies believe. The rapid palatal expansion technique has no interference in the lowering of the palate; otherwise, the results from T_0_ to T_2_ should not be increased, but decreased. As we can see in the results, the technique maintains stability in the palatal depth only, providing enlargement of the midpalatal suture. 

## CONCLUSIONS

According to the findings of this study, it is possible to conclude that RME offers a significant gain in the surface area of the palate and a significant increase in the intermolar width, facts that are directly related to the opening of the midpalatal suture. Therefore, the use of the Hyrax appliance was effective for the treatment of maxillary atresia in growing patients. 

There was no vertical alteration of the palate, since the depth of the palate did not show significant changes.

After a 6-month retention period with the Hyrax appliance, the transverse dimension of the maxilla and the surface area of the palate remained stable.

## References

[1] Haas AJ (1961). Rapid expansion of the maxillary dental arch and nasal cavity by opening the midpalatal suture. Angle Orthod.

[2] Haas AJ (1965). The treatment of maxillary deficiency by opening the midpalatal suture. Angle Orthod.

[3] Garrett BJ, Caruso JM, Rungcharassaeng K, Farrage JR, Kim JS, Taylor GD (2008). Skeletal effects to the maxilla after rapid maxillary expansion assessed with cone-beam computed tomography. Am J Orthod Dentofacial Orthop.

[4] Ghoneima A, Abdel-Fattah E, Eraso F, Fardo D, Kula K, Hartsfield J (2010). Skeletal and dental changes after rapid maxillary expansion a computed tomography study. Aust Orthod J.

[5] Baka ZM, Akin M, Ucar FI, Ileri Z (2015). Cone-beam computed tomography evaluation of dentoskeletal changes after asymmetric rapid maxillary expansion. Am J Orthod Dentofacial Orthop.

[6] Bishara SE, Staley RN (1987). Maxillary expansion clinical implications. Am J Orthod Dentofacial Orthop.

[7] Enlow HD (1982). Handbook of facial growth. Introductory concepts of the growth process.

[8] Woller JL, Kim KB, Behrents RG, Buschang PH (2014). An assessment of the maxilla after rapid maxillary expansion using cone beam computed tomography in growing children. Dental Press J Orthod.

[9] Cross DL, McDonald JP (2000). Effect of rapid maxillary expansion on skeletal, dental, and nasal structures a postero-anterior cephalometric study. Eur J Orthod.

[10] Ladner PT, Muhl ZF (1995). Changes concurrent with orthodontic treatment when maxillary expansion is a primary goal. Am J Orthod Dentofacial Orthop.

[11] Bazargani F, Feldmann I, Bondemark L (2013). Three-dimensional analysis of effects of rapid maxillary expansion on facial sutures and bones. Angle Orthod.

[12] Velazquez P, Benito E, Bravo LA (1996). Rapid maxillary expansion A study of the long-term effects. Am J Orthod Dentofacial Orthop.

[13] Lemieux G, Carey JP, Flores-Mir C, Secanell M, Hart A, Dietrich N (2014). Three-dimensional cephalometric superimposition of the nasomaxillary complex. Am J Orthod Dentofacial Orthop.

[14] Ballanti F, Lione R, Fanucci E, Franchi L, Baccetti T, Cozza P (2009). Immediate and post-retention effects of rapid maxillary expansion investigated by computed tomography in growing patients. Angle Orthod.

[15] Gracco A, Malaguti A, Lombardo L, Mazzoli A, Raffaeli R (2010). Palatal volume following rapid maxillary expansion in mixed dentition. Angle Orthod.

[16] Marini I, Bonetti GA, Achilli V, Salemi G (2007). A photogrammetric technique for the analysis of palatal three-dimensional changes during rapid maxillary expansion. Eur J Orthod.

[17] Lione R, Franchi L, Cozza P (2012). Does rapid maxillary expansion induce adverse effects in growing subjects. Angle Orthod.

[18] Primozic J, Baccetti T, Franchi L, Richmond S, Farcnik F, Ovsenik M (2013). Three-dimensional assessment of palatal change in a controlled study of unilateral posterior crossbite correction in the primary dentition. Eur J Orthod.

[19] Baumrind S, Carlson S, Beers A, Curry S, Norris K, Boyd R (2003). Using three-dimensional imaging to assess treatment outcomes in orthodontics a progress report from the University of the Pacific. Orthod Craniofac Res.

[20] Podesser B, Williams S, Crismani AG, Bantleon H-P (2007). Evaluation of the effects of rapid maxillary expansion in growing children using computer tomography scanning a pilot study. Eur J Orthod.

[21] Phatouros A, Goonewardene MS (2008). Morphologic changes of the palate after rapid maxillary expansion a 3-dimensional computed tomography evaluation. Am J Orthod Dentofacial Orthop.

[22] Lagravère MO, Heo G, Major PW, Flores-Mir C (2006). Meta-analysis of immediate changes with rapid maxillary expansion treatment. J Am Dent Assoc.

[23] Pangrazio-Kulbersh V, Wine P, Haughey M, Pajtas B, Kaczynski R (2012). Cone beam computed tomography evaluation of changes in the naso-maxillary complex associated with two types of maxillary expanders. Angle Orthod.

[24] Ramoglu SI, Sari Z (2010). Maxillary expansion in the mixed dentition rapid or semi-rapid?. Eur J Orthod.

